# A 30-Min Nucleic Acid Amplification Point-of-Care Test for Genital *Chlamydia trachomatis* Infection in Women: A Prospective, Multi-center Study of Diagnostic Accuracy

**DOI:** 10.1016/j.ebiom.2017.12.029

**Published:** 2018-01-10

**Authors:** E.M. Harding-Esch, E.C. Cousins, S.-L.C. Chow, L.T. Phillips, C.L. Hall, N. Cooper, S.S. Fuller, A.V. Nori, R. Patel, S. Thomas-William, G. Whitlock, S.J.E. Edwards, M. Green, J. Clarkson, B. Arlett, J.K. Dunbar, C.M. Lowndes, S.T. Sadiq

**Affiliations:** a Applied Diagnostic Research and Evaluation Unit, Institute for Infection and Immunity, St George's University of London, London SW17 0RE, UK; b Public Health England, National Infection Service, HIV/STI Department, Colindale, London NW9 5EQ, UK; c Solent Sexual Health, University of Southampton, UK; d The Starling Clinic, Musgrove Park Hospital, Taunton and Somerset NHS Foundation Trust, UK; e 56 Dean Street, Chelsea & Westminster Hospital NHS Foundation Trust, London, UK; f Sexual Health Hertfordshire Chelsea and Westminster NHS Foundation Trust, London SW10 9NH, UK; g Central London Community Healthcare NHS Trust, London SW1E 6QP, UK; h Atlas Genetics, Derby Court, Epsom Square, White Horse Business Park, Trowbridge, Wiltshire, BA14 0XG, UK; i Courtyard Clinic, St George's University Hospitals NHS Foundation Trust, London, UK, SW17 0QT, UK

**Keywords:** *Chlamydia trachomatis*, Rapid test, Point-of-care, Diagnostic accuracy, Performance evaluation, Risk factor

## Abstract

**Background:**

Rapid Point-Of-Care Tests for *Chlamydia trachomatis* (CT) may reduce onward transmission and reproductive sexual health (RSH) sequelae by reducing turnaround times between diagnosis and treatment. The io® single module system (Atlas Genetics Ltd.) runs clinical samples through a nucleic acid amplification test (NAAT)-based CT cartridge, delivering results in 30 min.

**Methods:**

Prospective diagnostic accuracy study of the io® CT-assay in four UK Genito-Urinary Medicine (GUM)/RSH clinics on additional-to-routine self-collected vulvovaginal swabs. Samples were tested “fresh” within 10 days of collection, or “frozen” at − 80 °C for later testing. Participant characteristics were collected to assess risk factors associated with CT infection.

**Results:**

CT prevalence was 7.2% (51/709) overall. Sensitivity, specificity, positive and negative predictive values of the io® CT assay were, respectively, 96.1% (95% Confidence Interval (CI): 86.5–99.5), 97.7% (95%CI: 96.3–98.7), 76.6% (95%CI: 64.3–86.2) and 99.7% (95%CI: 98.9–100). The only risk factor associated with CT infection was being a sexual contact of an individual with CT.

**Conclusions:**

The io® CT-assay is a 30-min, fully automated, high-performing NAAT currently CE-marked for CT diagnosis in women, making it a highly promising diagnostic to enable specific treatment, initiation of partner notification and appropriately intensive health promotion at the point of care.

## Highlights

• The io® CT assay's sensitivity is comparable to that of laboratory-based assays commonly used for *Chlamydia* detection.
• The specificity as shown is this study is at the lower end of the range reported for laboratory-based assays.
• The resulting positive predictive value, in this population, indicates that a targeted testing approach may be optimal.

Until now, there have been no ≤ 30-min point-of-care test (POCT) nucleic acid amplification tests (NAATs) on the market for detecting *Chlamydia trachomatis* (CT), despite cost-effectiveness analyses demonstrating they may improve Genito-Urinary Medicine (GUM)/Reproductive and Sexual Health (RSH) clinical care pathway efficiencies and patient outcomes. Our evaluation of the 30-min Atlas Genetics io® CT NAAT POCT, on vaginal swabs from women attending four GUM/RSH clinics in England, shows that the test's sensitivity (accurately detecting CT-positive infections) is similar to that of laboratory-based NAATs, and its specificity (accurately detecting CT-negative infections) is at the lower end of the laboratory-based NAAT range.

## 1 Introduction

Genital infection with *Chlamydia trachomatis* (CT) is a major public health challenge with over 200,000 CT diagnoses made in England alone in 2016, accounting for nearly half of all new sexually transmitted infection (STI) diagnoses that year (Public Health England, 2016). CT infection, which most commonly occurs in young people aged 15–24 years, goes undiagnosed in a large proportion of cases, is often asymptomatic in women (70%) and men (50%), and can lead to serious reproductive health morbidities, such as pelvic inflammatory disease, tubal infertility and ectopic pregnancy (O'Connell and Ferone, 2016). The treatment of new CT diagnoses alone was predicted to contribute to nearly a third of the estimated direct medical costs of treating new STI diagnoses in the UK in 2011 (Lucas, 2013).

Shortening the duration of infectiousness (between becoming infected and receiving effective treatment) of CT in at-risk individuals is key to curbing CT transmission (Huppert et al., 2010, Steen et al., 2009), while shortening the duration of infection can reduce reproductive health complications (Geisler et al., 2008, Haggerty et al., 2010). The time between testing and treatment can vary widely (Public Health England, 2014) meaning some patients will wait longer for results and therefore have increased risk of transmitting infection. Public health programs, such as the National Chlamydia Screening Programme (NCSP) in England, set standards for the time to treatment to guide services and reduce variation in care, thereby bringing the average time to treatment down. Nucleic acid amplification tests (NAATs) are recommended for routine diagnosis of CT infections by national and international guidelines (Nwokolo et al., 2016, World Health Organisation, 2015, Centers for Disease Control and Prevention, 2015) because of their high sensitivity and specificity, ability to deliver high volume testing and their relatively low cost. In addition to these advantages, NAAT-based point-of-care tests (POCTs), which enable patients to be tested and treated within the same clinical visit, have the potential to reduce the time to treatment and improve patient care (Turner et al., 2014, Adams et al., 2014, Harding-Esch et al., 2017b). Rapid NAATs, such as the Cepheid CT/*Neisseria gonorrhoeae* (NG) GeneXpert assay, have equivalent performance characteristics to traditional lab-based NAATs (Gaydos et al., 2013), but the 90 min test-run time is too long for the test to be considered a POCT in many healthcare settings (Harding-Esch et al., 2017b, Badman et al., 2016, Natoli et al., 2015). In addition, studies have shown a significant proportion of service users are unwilling to wait more than around 20 min for test results (Atkinson et al., 2016, Rompalo et al., 2013). There is therefore a need for more rapid (≤ 30 min) NAAT-based POCTs to aid accurate and specific diagnoses during one clinic visit.

The Atlas Genetics io® platform is a 30-min NAAT, single module system. Clinical samples, such as swab eluate, are transferred directly into a microfluidic cartridge for the diagnosis of genital CT in females. The io® was designed to require no user training; an instrument manual and test Instructions For Use are provided, and the instrument walks the user through the test set-up steps on the on-board touchscreen. The dimensions of the instrument are 275 mm × 384 mm × 277 mm (w x d x h).

The objective of this study was to conduct a performance evaluation of the io® CT assay using genital samples collected from females attending UK Genito-Urinary Medicine (GUM)/Reproductive and Sexual Health (RSH) clinics. Secondary objectives were to assess factors associated with being CT positive to inform on how to potentially implement the io® CT assay in clinical pathways.

## 2 Materials and Methods

The study was conducted in accordance with the Declaration of Helsinki, and was approved by the London Bridge Research Ethics Committee (REC reference 13/LO/0691, IRAS reference 126,709). All participants gave written, informed consent before taking part in the study. Participants gave informed consent for anonymized results to be used by the researchers for publication in medical journals and presentation at scientific meetings. This manuscript was written following STARD (Standards for the Reporting of Diagnostic accuracy) guidelines (Bossuyt et al., 2015) (supplemental files 1 and 2).

Four GUM/RSH clinics in London (Dean Street Clinic, Chelsea and Westminster Hospital NHS Trust), Taunton (Musgrove Park Hospital, Taunton and Somerset NHS Foundation Trust), Portsmouth (Solent NHS Trust) and Stevenage (Kingsway Sexual Health Service, Central London Community Healthcare NHS Trust) were selected for participant recruitment, which took place between June 2015 and March 2016.

### 2.1 Participants

Symptomatic and asymptomatic female participants were recruited prospectively and were considered eligible for the evaluation if they were: attending the participating GUM/RSH clinics and having a routine CT NAAT; aged 16 years or over; able to provide written informed consent; able and willing to provide an additional to routine self-collected vulvovaginal swab (SCVS). We defined participants to be potentially symptomatic for CT if they presented with any of the following: genital itching, discharge (clear or cloudy liquid from the vagina), pain/burning when urinating, needing to pass urine more often than usual, pain deep inside the vagina when having sex, pain just inside or around the vagina when having sex, bleeding after sex, bleeding in between periods or pelvic abdominal pain. Participants were recruited following provision of written informed consent and given a unique participant identifier (participant ID). Routine clinical and demographic data were recorded prospectively by a delegated clinical staff member.

Based on a conservative estimate of the sensitivity of the io® CT assay of 92% (95% confidence interval (95%CI); 81-97) and a mean CT positivity rate of 7.5% in GUM/RSH clinics (Hamish Mohammed, Public Health England (PHE), personal communication) the target sample size was 750 females to obtain 50 CT positive female samples. Recruitment of participants formed a convenience sample, with clinics preferentially recruiting participants who they judged were more likely to be CT positive (e.g. CT-positive sexual partner) (Wiesenfeld, 2017) in order to increase the likelihood of meeting the 50 CT positive target.

### 2.2 Specimen Collection

Following collection of the clinic's routine vulvovaginal swab for CT/NG NAAT diagnosis, an SCVS (Copan eNAT® Collection and Preservation System) was provided for the study. If the participant was having a vaginal examination, she was asked to take the SCVS sample before the examination. Routine vulvovaginal swabs were processed as per clinical protocol, and the additional SCVS samples stored at room temperature, initially in clinic for a maximum of six days, and then transferred at between 2 and 8 °C to the Applied Diagnostic Research and Evaluation Unit (ADREU) laboratories at St George's, University of London (SGUL). Upon receipt, samples were aliquoted as follows: 600 μl for testing with the io® CT assay; 300 μl for discrepant result testing, if necessary (see below); and any remainder (approximately 1 ml) for repeat testing as required. This ensured there was sufficient volume for testing on each platform, as specified in manufacturers' instructions. Samples received with insufficient volume for both an initial and discrepant sample aliquot were excluded from the study. Samples were either tested “fresh” within ten days of collection (stored refrigerated (2-8 °C) at SGUL) or immediately frozen at − 80 °C. These samples are subsequently termed “fresh” or “frozen” respectively through the manuscript. Aliquots for discrepant and repeat testing were also immediately frozen at —80C. “Frozen” samples were defrosted for a minimum of 30 min and tested within two hours.

The research sample and case report form data were linked to clinical results using the unique participant IDs. Once all data were matched and data verification complete, the temporarily list linking participant and clinical identifiers was destroyed, thus anonymizing the data.

### 2.3 Test Methods

Four io® systems were used to test the samples between September 2015 and September 2016. The median number of days between sample collection and io® CT assay testing for “fresh” samples was seven days. All samples were tested within 10 days, except for one tested at 13 days. For “frozen” samples (frozen on receipt and thawed for 30 min before testing), the minimum and maximum number of days between sample collection and testing were 12 and 452, with a median of 210 days. io® CT assay cartridges were kept refrigerated prior to use. Positive and negative io® CT assay control cartridges were run on each io® system daily before sample testing to validate the system.

A fixed volume pipette, packaged with each cartridge, was used to withdraw and transfer 500 μl of sample to a port on the cartridge, which was then loaded onto the io® system. The participant ID was scanned into the system and testing started via a touch-screen control. Within the io® system, without any user involvement, an automated processing of the sample took place in a series of microfluidic channels and chambers, comprising a number of steps that included extraction of CT DNA, amplification of a small specific segment of DNA by Polymerase Chain Reaction (PCR) and detection of the amplified DNA using a ferrocene derivative electrochemically-labeled DNA probe.

In all cases, results were delivered in 30 min as either ‘CT detected’, ‘CT not detected’ or ‘invalid’. If a sample returned an invalid result, a repeat test was performed on a new cartridge. If invalid a second time, the final result was recorded as invalid. ADREU laboratory staff carrying out the testing on the io® system were blind to participant clinical information and the clinic CT/NG NAAT results.

### 2.4 Estimating Diagnostic Accuracy

For all samples, the initial comparator test used was the CE-marked Becton Dickinson (BD) ProbeTec™ Qx CT/GC assay (Oxford, UK), run on the BD Viper analyzer, as this was the routine CT/NG NAAT used at all participating GUM/RSH clinics. Those conducting the initial comparator test were blind to clinical information and io® CT assay results. The io® CT assay results were compared to the initial comparator test results by the ADREU study Coordinator, and any discordant results identified.

We defined the reference standard (Bossuyt et al., 2015) as the initial comparator test result when in agreement with the io® CT assay result. If the io® CT assay result did not agree with the initial comparator test result, a further test with the CE-marked QIAgen Artus® *C. trachomatis* Plus RG PCR kit (Manchester, UK), run on the Qiagen Rotor-Gene Q 2plex HRM PCR thermocycler, was performed according to manufacturer's instructions. In these cases the reference standard was defined as the resolved result when two out of three of io® CT assay, initial reference test and Artus CT assay results were in agreement. This discrepant analysis approach was employed as a result of budgetary and time constraints. The Artus CT assay was selected as it was the assay for discrepant testing in a previous io® CT assay diagnostic evaluation (Pearce et al., 2015).

### 2.5 Statistical Analysis

Data from the io® system were transcribed manually onto the study database. Data cleaning and validation were performed independently and separately at SGUL and at PHE. Any discrepancies were resolved through checking the original data with the clinics. Data were analyzed at PHE using Stata (StataCorp LP v13.1). Missing data were verified, and all initial comparator test results double-checked, with each clinic. Participants with either a missing io® CT assay or initial comparator test result were excluded from analyses.

Diagnostic accuracy metrics (sensitivity, specificity, positive (PPV) and negative (NPV) predictive values) and their 95% CIs (binomial exact) were calculated. A two-sample chi-squared test was performed to compare results by symptomatic and asymptomatic status and sample storage method (“frozen” versus unfrozen (“fresh”)) (Table 1). CT prevalence was assessed at the clinic population level using the GUM Clinical Activity Dataset (GUMCAD) (Public Health England, 2013) for the clinics involved over the study period to inform if there had been any bias in recruitment. We also compared the impact of the performance measures of the io® CT assay on PPV and NPV using national GUMCAD prevalence data (Hamish Mohammed, PHE, personal communication) (high prevalence setting) as well as from Natsal-3 (National Survey of Sexual Attitudes and Lifestyles) (Sonnenberg et al., 2013) (low prevalence setting), focusing on women aged 16–24 years. Natsal-3 is a population-based prevalence survey representing all adults resident in the UK between the ages of 16–74 years.

**Table 1:** Diagnostic accuracy of the io® CT assay when compared with the reference standard^a^.

	All	Symptomatic	Asymptomatic	Symptomatic vs asymptomatic p-value^b^	“Fresh”	“Frozen”	“Fresh” vs “frozen” p-value^b^
Prevalence	7.2 (5.4–9.3) (51/709)	11 (6.0–18.1) (13/118)	6.4 (4.6–8.7) (38/591)	0.106	9.3 (6.1–13.3) (26/281)	5.8 (3.8–8.5) (25/428)	0.111
Sensitivity	96.1 (86.5–99.5) (49/51)	100.0 (75.3–100.0) (13/13)	94.7 (82.3–99.4) (36/38)	0.906	96.2 (80.4–99.9) (25/26)	96.0 (79.6–99.9) (24/25)	0.997
Specificity	97.7 (96.3–98.7) (643/658)	98.1 (93.3–99.8) (103/105)	97.6 (96.0–98.7) (540/553)	0.976	96.5 (93.4–98.4) (246/255)	98.5 (96.8–99.5) (397/403)	0.854
PPV	76.6 (64.3–86.2) (49/64)	86.7 (59.5–98.3) (13/15)	73.5 (58.9–85.1) (36/49)	0.706	73.5 (55.6–87.1) (25/34)	80.0 (61.4–92.3) (24/30)	0.824
NPV	99.7% (98.9–100.0) (643/645)	100.0 (96.5–100.0) (103/103)	99.6 (98.7–100.0) (540/542)	0.981	99.6 (97.8–100.0) (246/247)	99.7 (98.6–100.0) (397/398)	0.989

CT, *Chlamydia trachomatis*; PPV, Positive Predictive Value; NPV, Negative Predictive Value.

a Values are percentages (95% confidence intervals) (numbers).

b Study prevalence of CT and performance characteristics (sensitivity, specificity, PPV and NPV) were compared between symptomatic and asymptomatic participants and between “fresh” and “frozen” samples using the Pearson Chi-squared test for comparing two proportions.

Univariate logistic regression analysis of risk factors associated with CT infection (as defined by the reference standard) was conducted. Factors considered significant (p < 0.05) were included in a multivariate analysis employing a forward step-wise approach, with age and clinic considered a priori risk factors. The same process was used to determine any factors associated with an invalid, or discrepant, io® CT assay result. Factors included in the analyses were: participant age, sexual orientation, having taken medication that would be active against CT infection in the last 6 weeks, being a contact of a CT-positive individual, having had an STI in the last 12 months, whether currently menstruating, whether symptomatic for CT infection, and clinic attended. In the invalid io® CT assay result logistic regression analysis, clinic routine CT NAAT and NG NAAT results were also included as explanatory variables.

### 2.6 Data Statement

The datasets used and/or analyzed during the current study are available from the corresponding author on reasonable request.

## 3 Results

### 3.1 Participants and Sites

**Fig. 1:**
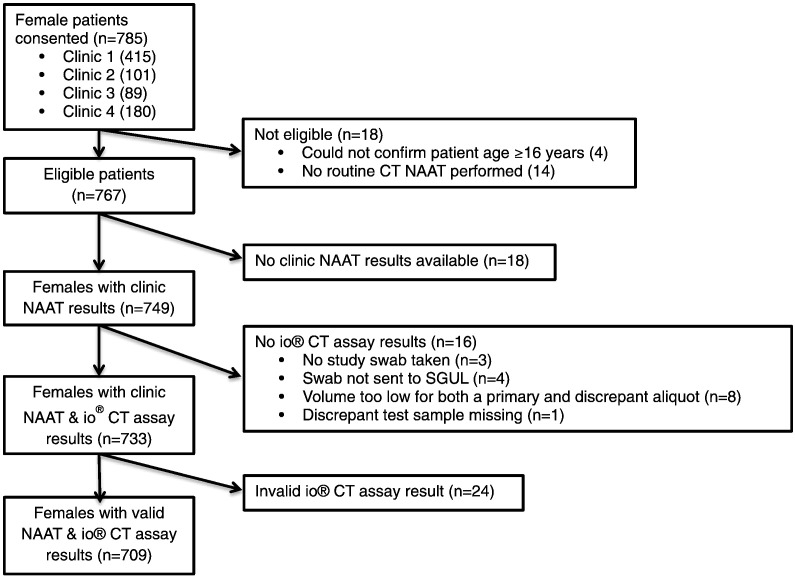
Flow chart summarizing patient recruitment and sample collection results availability. CT, *Chlamydia trachomatis*; NAAT, Nucleic Acid Amplification Test. Flow diagram showing total number of eligible participants who consented to the study, ending with the total number of participants included in the final analyses. Samples were excluded where the participant did not meet the study eligibility criteria (n = 18); that did not have a clinic NAAT result available (n = 18), that were not tested on the io® CT assay (n = 16); or that had a final invalid result by the io® CT assay (n = 24).

A total of 785 female participants were recruited from the different clinics (Fig. 1). 76 participants were excluded from the final analyses, conducted on 709 (90.3%) participants, for reasons including not fulfilling eligibility criteria, missing initial reference test data (clinic BD Viper CT/NG NAAT), and final invalid io® CT assay results. The overall CT prevalence according to the reference standard among the samples tested was 7.2% (51/709; 95%CI 5.4–9.3) with no difference between “fresh” and “frozen” samples (Table 1). Baseline participant demographic and clinical characteristics are presented in Table 2.

**Table 2:** Risk factor analysis for being CT positive^a^.

			Univariate analysis			Multivariate analysis^c^		
Characteristic	No. of participants	No. (%) with CT^b^	OR	95%CI	*P*-value	OR	95%CI	P-value
Age	709	51 (7.2)	0.94	0.89–0.99	0.01	0.95	0.90–1.00	0.06
Clinic								
1	387	34 (8.8)	1			1		
2	92	3 (3.3)	0.35	0.11–1.17	0.09	0.36	0.10–1.29	0.12
3	72	2 (2.8)	0.30	0.07–1.26	0.10	0.31	0.07–1.43	0.13
4	158	12 (7.6)	0.85	0.43–1.69	0.65	0.47	0.20–1.07	0.07
Contact of a CT positive								
No	617	26 (4.2)	1			1		
Yes	48	21 (43.8)	17.68	8.85–35.33	< 0.001	17.33	8.28–36.27	< 0.001
Not known	44	4 (9.1)	2.27	0.76–6.83	0.14	2.94	0.91–9.45	0.07
Taken CT-active medication in last 6 weeks								
No	669	47 (7.0)	1					
Yes	40	4 (10.0)	1.47	0.50–4.31	0.48			
Symptomatic								
No	591	38 (6.4)	1					
Yes	118	13 (11.0)	1.8	0.93–3.50	0.08			
STI last year								
No	641	41 (6.4)	1			1		
Yes	68	10 (14.7)	2.52	1.20–5.30	0.01	2.09	0.87–4.99	0.10
Currently menstruating								
No	634	46 (7.3)	1					
Yes	71	4 (5.6)	0.76	0.27–2.19	0.62			
Has sex with^d^								
Men	665	47 (7.1)	1					
Women	20	1 (5.0)	0.70	0.09–5.28	0.72			
Both	23	3 (13.0)	1.98	0.57–6.88	0.29			

CT, *Chlamydia trachomatis*; OR, Odds ratio; 95% CI, 95% Confidence Interval; STI, Sexually transmitted infection.

a For each characteristic the number of participants and the proportion of these with a CT infection are shown.

b CT positive defined as reference standard: either positive initial comparator test result (when in agreement with io® CT assay result); or positive by at least two of three of the initial comparator test, io® CT assay and, Artus CT assay).

c Adjusted for age, clinic, contact status and STI in the last year (age and clinic considered a priori risk factors and included in all models).

d Sexual orientation unknown for one participant.

### 3.2 Performance of the Io® CT Assay

A number of tests (n = 24/733; 3.3%) reported an invalid result on the first run and 100% of these reported an invalid result on a second. On review of the electrochemical trace data, none of the invalids had an internal control (IC) peak, indicating that the IC DNA did not amplify. Of the 24 invalid results, 20 were negative and four were positive for CT by the clinic routine CT/NG NAAT. In the logistic regression analysis, there were no factors associated with the io® CT assay result being invalid or discrepant.

684/709 (96.5%) io® CT assay results agreed with the initial comparator test result. For the remaining 25 samples that had a discordant result between the io® CT assay and initial comparator test, the Artus CT assay (used for discrepant testing) agreed with 8 io® CT assay results (4/19 io® CT assay positives and 4/6 io® CT assay negatives). Of the 281 samples tested “fresh” and the 428 samples tested from “frozen”, 14 (5.0%) and 11 (2.6%) were discrepant, respectively. The minimum and maximum number of days between sample collection and Artus CT assay testing for “fresh” samples were 13 and 435, with a median of 23.5 days. For “frozen” samples, the minimum and maximum number of days between sample collection and testing were 56 and 521, with a median of 452. The same technician performed both io® CT and Artus CT assay testing.

The resulting sensitivity, specificity, PPV and NPV overall, compared with the reference standard, were 96.1% (95%CI 86.5-99.5), 97.7% (95%CI 96.3-98.7), 76.6% (95%CI 64.3-86.2) and 99.7% (95%CI 98.9-100.0) respectively (Table 1). There were no significant differences in the performance of the io® CT assay in any of the diagnostic accuracy measures between symptomatic and asymptomatic participants, or between “fresh” and “frozen” SCVS samples run on the io® system (Table 1).

The study CT prevalence of 7.2% was higher than the 5.2% (p = 0.0182) prevalence of all female patients routinely tested for CT attending the study clinics over the study period, determined by clinic GUMCAD data (Public Health England, 2013). National CT prevalence data for females from GUMCAD (Hamish Mohammed, PHE, personal communication), and Natsal-3 (Sept 2010-Aug 2012) (Sonnenberg et al., 2013) were 6.7% and 3.1%, respectively. PPVs for these data sets were 74.4% (95%CI 65.8-81.5) and 57.7% (95%CI 47.3-67.4) respectively. NPVs were comparable to the study NPV.

### 3.3 Risk Factors for Being CT Positive

Factors associated with being CT positive in univariate logistic regression analysis were young age, being a sexual contact of a CT positive individual and having had an STI in the last year (Table 2). In multivariate analysis, only being a sexual contact of CT remained as an independent risk factor.

## 4 Discussion

National and international guidelines recommend laboratories use NAATs for the diagnosis of CT infections due to their superior performance compared with other diagnostic technologies (Nwokolo et al., 2016, World Health Organisation, 2015, Centers for Disease Control and Prevention, 2015). Performance evaluations for US Food and Drug Administration (FDA) approval use a Patient Infection Status (PIS) or composite gold standard study design, with data for some of the most commonly used laboratory-based NAATs on self-collected vaginal swabs indicating sensitivities between 96.5%–98.4% and specificities between 95.6%–99.2% (Hologic, 2016b, Hologic, 2016a, Abbott, 2010, Taylor et al., 2011). The Cepheid GeneXpert CT/NG rapid NAAT, which is not a traditional laboratory-based NAAT, has a reported sensitivity and specificity of 98.7% (95%CI 93.1–100) and 99.4% (95%CI 98.9-99.7) using a PIS reference standard (Gaydos et al., 2013). We however employed a discrepant analysis approach for our study, and published evaluations of laboratory-based NAATs for CT detection using vaginal swabs using this approach report sensitivities of 80.4%–100.0%, and specificities of 99.5%–100.0% (Geelen et al., 2013, de Waaij et al., 2015, Masek et al., 2009). The io® CT POCT, as evaluated in our study, thus has a comparable sensitivity but potentially lower specificity than these laboratory-based NAATs. However, a previous evaluation of the io® CT assay, also employing a discrepant analysis approach, indicated a specificity comparable to those reported for laboratory-based NAATs and higher than in our study, of 99.0% (USA) (Widdice et al., 2017). This variation in performance measures achieved supports the importance of conducting diagnostic accuracy studies in different settings and populations (TDR Diagnostics Evaluation Expert Panel et al., 2010).

A test's PPV is directly affected by the CT prevalence in the population tested. To compare how the laboratory-based NAATs evaluated by discrepant analysis would perform in our study population, we calculated their PPVs using our study prevalence of 7.2%. The resulting PPVs ranged between 93.9% (95%CI 84.6–98.8) and 100% (95%CI 93.0–100.0). The io® CT assay PPV in our study of 76.6% (95%CI 64.3–86.2) is at the lower end of the range. When applying the sensitivity and specificity estimates from the previous io® CT assay diagnostic accuracy evaluation (Widdice et al., 2017) to our study prevalence, a PPV of 87.9% (95%CI 75.5–94.7) was achieved. We also assessed how the io® CT assay would perform in high (GUMCAD) and low (Natsal) CT prevalence settings by calculating how the predictive values would change based on the prevalence data reported for these settings. PPVs were 74.4% and 57.7% for GUMCAD and Natsal respectively, and NPVs were 99.7% and 99.9% respectively. Previous British Association for Sexual Health and HIV (BASHH) guidelines from 2010 (Clinical Effectiveness Group British Association for Sexual Health and HIV, 2010) stated that testing platforms must have a PPV of over 90%. This is no longer mentioned in current BASHH guidelines (BASHH, 2015), possibly because previous guidelines were written at a time when performance characteristics for laboratory-based NAATs were variable and when POCT NAATs were not available. The way in which the io® CT assay is best implemented in clinical pathways as a POCT in view of these PPV results should be considered, for example assessing patient CT infection risk to increase CT positivity in the tested population.

Consequently, risk factor analyses may be helpful in targeting who to test with the io® CT assay. Previous work in UK GUM/RSH clinics has indicated that younger age (< 20), more than one (concurrent) sexual partner, black ethnicity and smoking are independent risk factors for CT in women (Radcliffe et al., 2001). In our analysis, risk factors included younger age, having had an STI in the last year and being a sexual contact of someone diagnosed with CT in univariate analyses, although only the latter remained an independent risk factor in the multivariate analysis. Being a sexual contact of an individual with an STI is reported to be a risk factor for CT infection in studies from Europe and the US (Wiesenfeld, 2017). It is important to consider that in other populations or settings these risk factors may be different (Sonnenberg et al., 2013, Wiesenfeld, 2017, Aghaizu et al., 2014), and targeted testing might need to be adjusted accordingly. This targeted-patient approach is further supported by the io® system's current single-modular platform design, which has the potential for multiple systems to be placed in a clinic at any one time.

Equally, there are practical implications of the io® CT assay's 3.3% failure rate, which although consistent with that of the GeneXpert CT/NG assay on first attempt (Gaydos et al., 2013), did not improve after repeat testing, suggesting that following an initial invalid result, patients would need to be recalled to provide a new sample. The fact that all invalid results were associated with IC DNA not amplifying points to PCR inhibition, possibly from inhibitors in the sample, which the io® CT assay sample purification process may not adequately have removed. This may have implications for the cost-effectiveness of deploying this test, and with no factors predicting likelihood of an invalid result, it is not possible to factor in adjustments to sample collection pathways to mitigate potential invalid test results.

Sampling from different UK GUM/RSH clinics enabled us to both target high-risk patients to achieve the positivity required for the study and capture different populations that are more representative of the GUM/RSH clinic attendees for the whole of the UK than would have been possible with a single-site study. The operators performing the io® CT assay testing were blind to the initial comparator test results, and vice versa, as well as to participant clinical and demographic characteristics, and data analysis was conducted independently at PHE. All participating clinics used the same routine clinic NAAT to provide a CT diagnosis for participants ensuring consistency across the study. However, only one sample type for one anatomical site was evaluated. Whereas vulvovaginal swabs are routinely used in GUM/RSH clinics for NAAT diagnosis of urogenital CT infection, *endo*-cervical and urine samples are also commonly used (Gaydos et al., 2013, Van Der Pol et al., 2012), and there is increasing evidence for the importance of extra-genital sampling in women (Chandra et al., 2016, Koedijk et al., 2012). When analyzing GUMCAD data for the study clinics during the study period, the CT prevalence of all women being tested for was 5.2%, lower than our participant 7.2% prevalence. Although this indicates a recruitment bias, probably because patients considered more likely to be CT-positive (e.g. CT-positive sexual partner (Wiesenfeld, 2017)) were approached to participate in order to meet our 50 CT-positive sample size, there is no reason to suspect that this would have affected our estimates of the io® CT assay's sensitivity and specificity.

We employed discrepant testing to define our reference standard if the io® CT assay result did not agree with that of the initial comparator test, rather than a composite gold standard approach where at least two reference tests are used together (typically searching for a different target), and a clear definition of a positive and a negative is provided (Hess et al., 2012, Alonzo and Pepe, 1999). Discrepant testing is known to introduce an initial bias towards the index test (io® CT assay) (Hadgu, 1999, McAdam, 2000). A better reference standard, decided by, for example, three independent reference tests against which the index test is compared, would have provided a more accurate estimate of performance of the io® CT assay (Alonzo and Pepe, 1999). However, this was not logistically possible within our study. Furthermore, it was not possible to test the samples further to investigate potential causes of the discrepancy, as there was not enough sample remaining. However, none of the factors included in our regression analysis were found to be associated with obtaining a discrepant result.

Another limitation relates to the method for the discrepant testing. The same technician performed both the io® CT and Artus CT assay testing and so were not blind to the Artus or routine clinic NAAT results, which could have led to bias. However this is unlikely as manufacturers' instructions were followed and the assay provides an objective result. In addition, the Copan eNat 6 month storage limit specified in the eNAT product specifications (COPAN, 2014) was not met for 11 (five “fresh”, six “frozen”) of the 25 discrepant samples. This is because delays in the verification process of discrepancy between the io® CT assay and routine clinic NAAT result meant that data were not always immediately available. It is possible that this may have led to DNA degradation, which could have resulted in some io® CT assay positives being misclassified as false-positives, or io® CT assay negatives misclassified as true-negatives. However, it has been shown that CT DNA in clinical SCVS does not significantly degrade within two years (van Dommelen et al., 2013), and we expect our samples to have been stable particularly as they were stored at -80 °C, as opposed to the − 20 °C noted in the eNAT specifications. In a sensitivity analysis, where we assumed that all Artus CT assay negatives were in fact positive, the sensitivity, specificity, PPV and NPV of the io® CT assay were 94.2% (49/52, 95%CI 84.1–98.8), 98.6% (648/657, 95%CI 97.4–99.4), 84.4% (49/58, 95%CI 72.6–92.7) and 99.5% (648/651, 95%CI 98.7–99.9), respectively. Of note, this sensitivity analysis results in the upper 95% CIs exceeding 99% for specificity and 90% for PPV.

The io® CT assay has been CE-marked and licensed for use in Europe in women only (Atlas Genetics Ltd, 2016). Future research is required to evaluate its performance in different settings, as well as in male participants. Although previous modelling work has demonstrated that a CT-only POCT could have an epidemiological impact and be cost-saving at the local level (Harding-Esch et al., 2015), dual CT/NG NAAT testing is most commonly employed in GUM/RSH settings in the UK. Therefore, further work is needed to assess the feasibility, acceptability and cost-effectiveness of POCTs for CT in GUM/RSH clinic settings (Harding-Esch et al., 2017a). Atlas Genetics Ltd. is currently developing a dual CT/NG assay for both male and female samples, which could overcome the limitations of a single-pathogen test.

We have shown that the io® CT assay is a 30-min, fully automated, high-performing NAAT currently CE-marked for CT diagnosis in women, making it a highly promising diagnostic to enable specific treatment, initiation of partner notification and appropriately intensive health promotion at the point of care. Future research is required to evaluate the io® CT assay's acceptability by clinicians and patients in GUM/RSH clinics, impact on clinical pathways and patient management, and cost-effectiveness.

## Appendix A Supplementary data

Supplementary material Image 1
