# Supplementary material for: A 30-Min Nucleic Acid Amplification Point-of-Care Test for Genital Chlamydia trachomatis Infection in Women: A Prospective, Multi-center Study of Diagnostic Accuracy
Source: eBioMedicine. 2018 Jan 10;28:120–7. doi: 10.1016/j.ebiom.2017.12.029 (PMC5897918; doi:10.1016/j.ebiom.2017.12.029)

**Figure 1. Flow chart summarizing patient recruitment and sample collection results availability**

Female patients consented (n=785)

- Clinic 1 (415)
- Clinic 2 (101)
- Clinic 3 (89)
- Clinic 4 (180)

Eligible patients (n=767)

Females with clinic NAAT results (n=749)

Not eligible (n=18)

- Could not confirm patient age ≥16 years (4)
- No routine CT NAAT performed (14)

No clinic NAAT results available (n=18)

No io® CT assay results (n=16)

- No study swab taken (n=3)
- Swab not sent to SGUL (n=4)
- Volume too low for both a primary and discrepant aliquot (n=8)
- Discrepant test sample missing (n=1)

Females with clinic NAAT & io^®^ CT assay results (n=733)

Females with valid NAAT & io® CT assay results (n=709)

Invalid io® CT assay result (n=24)

CT, *Chlamydia trachomatis*; NAAT, Nucleic Acid Amplification Test

Flow diagram showing total number of eligible participants who consented to the study, ending with the total number of participants included in the final analyses. Samples were excluded where the participant did not meet the study eligibility criteria (n=18); that did not have a clinic NAAT result available (n=18), that were not tested on the io® CT assay (n=16); or that had a final invalid result by the io® CT assay (n=24).

**Supplemental File 1. STARD checklist** (Bossuyt *et al.*, 2015)**.**

|  | **Section & Topic** | **No** | **Item** | **Reported on page #** |
| --- | --- | --- | --- | --- |
|  |  |  |  |  |
|  | **TITLE OR ABSTRACT** |  |  |  |
|  |  | **1** | Identification as a study of diagnostic accuracy using at least one measure of accuracy  (such as sensitivity, specificity, predictive values, or AUC) |  |
|  | **ABSTRACT** |  |  |  |
|  |  | **2** | Structured summary of study design, methods, results, and conclusions  (for specific guidance, see STARD for Abstracts) |  |
|  | **INTRODUCTION** |  |  |  |
|  |  | **3** | Scientific and clinical background, including the intended use and clinical role of the index test |  |
|  |  | **4** | Study objectives and hypotheses |  |
|  | **METHODS** |  |  |  |
|  | *Study design* | **5** | Whether data collection was planned before the index test and reference standard  were performed (prospective study) or after (retrospective study) |  |
|  | *Participants* | **6** | Eligibility criteria |  |
|  |  | **7** | On what basis potentially eligible participants were identified  (such as symptoms, results from previous tests, inclusion in registry) |  |
|  |  | **8** | Where and when potentially eligible participants were identified (setting, location and dates) |  |
|  |  | **9** | Whether participants formed a consecutive, random or convenience series |  |
|  | *Test methods* | **10a** | Index test, in sufficient detail to allow replication |  |
|  |  | **10b** | Reference standard, in sufficient detail to allow replication |  |
|  |  | **11** | Rationale for choosing the reference standard (if alternatives exist) |  |
|  |  | **12a** | Definition of and rationale for test positivity cut-offs or result categories  of the index test, distinguishing pre-specified from exploratory |  |
|  |  | **12b** | Definition of and rationale for test positivity cut-offs or result categories  of the reference standard, distinguishing pre-specified from exploratory |  |
|  |  | **13a** | Whether clinical information and reference standard results were available  to the performers/readers of the index test |  |
|  |  | **13b** | Whether clinical information and index test results were available  to the assessors of the reference standard |  |
|  | *Analysis* | **14** | Methods for estimating or comparing measures of diagnostic accuracy |  |
|  |  | **15** | How indeterminate index test or reference standard results were handled |  |
|  |  | **16** | How missing data on the index test and reference standard were handled |  |
|  |  | **17** | Any analyses of variability in diagnostic accuracy, distinguishing pre-specified from exploratory |  |
|  |  | **18** | Intended sample size and how it was determined |  |
|  | **RESULTS** |  |  |  |
|  | *Participants* | **19** | Flow of participants, using a diagram |  |
|  |  | **20** | Baseline demographic and clinical characteristics of participants |  |
|  |  | **21a** | Distribution of severity of disease in those with the target condition |  |
|  |  | **21b** | Distribution of alternative diagnoses in those without the target condition |  |
|  |  | **22** | Time interval and any clinical interventions between index test and reference standard |  |
|  | *Test results* | **23** | Cross tabulation of the index test results (or their distribution)  by the results of the reference standard |  |
|  |  | **24** | Estimates of diagnostic accuracy and their precision (such as 95% confidence intervals) |  |
|  |  | **25** | Any adverse events from performing the index test or the reference standard |  |
|  | **DISCUSSION** |  |  |  |
|  |  | **26** | Study limitations, including sources of potential bias, statistical uncertainty, and generalisability |  |
|  |  | **27** | Implications for practice, including the intended use and clinical role of the index test |  |
|  | **OTHER INFORMATION** |  |  |  |
|  |  | **28** | Registration number and name of registry |  |
|  |  | **29** | Where the full study protocol can be accessed |  |
|  |  | **30** | Sources of funding and other support; role of funders |  |
|  |  |  |  |  |

**Supplemental File 2. STARD flow diagram** (Bossuyt *et al.*, 2015)**.**


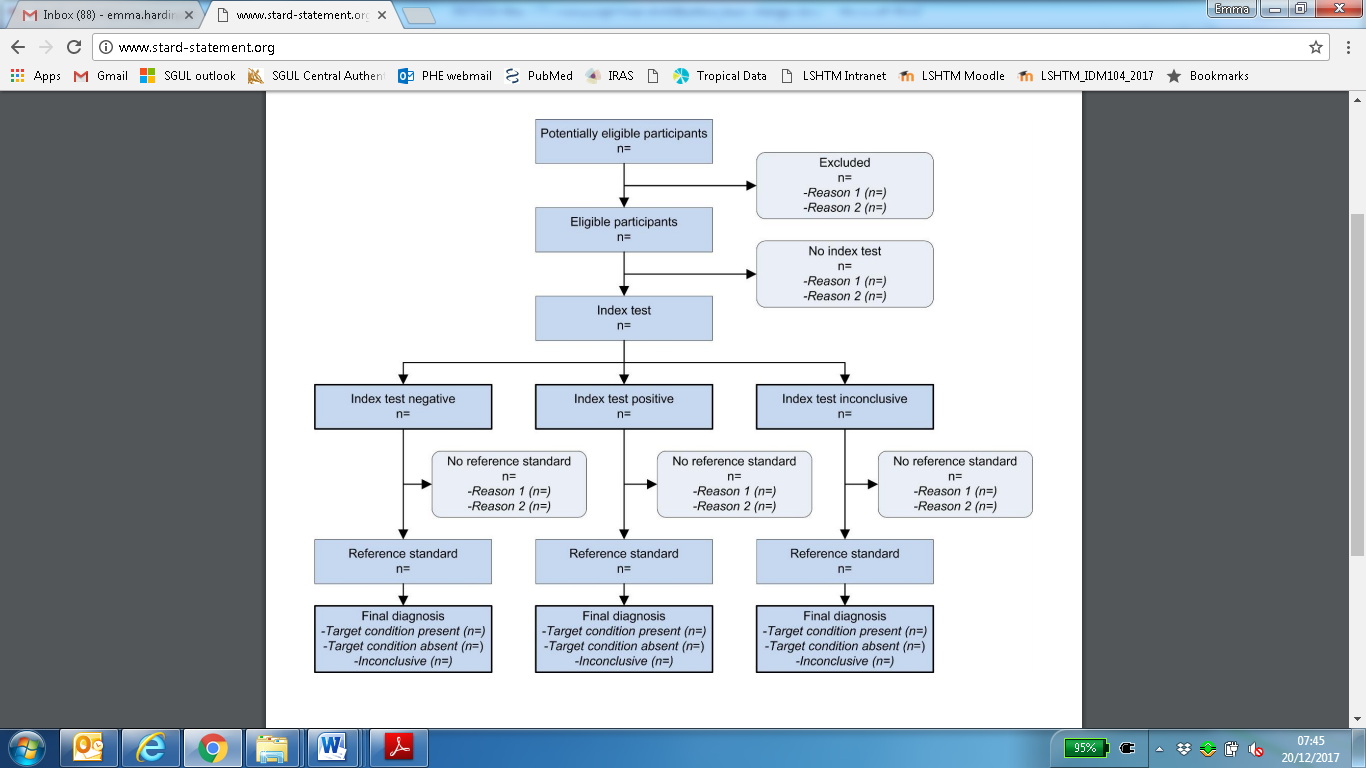

Supplement: Supplementary file 1 — Supplementary material [file mmc1.docx]
